# Effect of Active Oxygen Fluid (Blue®m) as a Root Canal Irrigant Against Enterococcus Faecalis

**DOI:** 10.3290/j.ohpd.b5740308

**Published:** 2024-09-12

**Authors:** Reem Barakat, Rahaf Almohareb, Arwa Alshahri, Nardeen Khawaji, Sarah Almufrij, Dhuha Alsuwaid, Fatma Alshehri

**Affiliations:** a Consultant Assistant Endodontics, Dental Clinics Department, King Abdullah bin Abdulaziz University Hospital, Princess Nourah Bint Abdulrahman University, Box 84428, Riyadh, 11671, Saudi Arabia. Conceptualisation, methodology, software, validation, formal analysis, review and editing, supervision, project administration.; b Associate Professor Endodontics, Department of Clinical Dental Sciences, College of Dentistry, Princess Nourah Bint Abdulrahman University P.O. Box 84428, Riyadh 11671, Saudi Arabia. Conceptualisation, methodology, validation, review and editing, funding acquisition.; c Dental Intern, College of Dentistry, Princess Nourah bint Abdulrahman University, P.O. Box 84428, Riyadh 1167, Saudi Arabia. Investigation, resources, writing (original draft preparation).; d Dental Intern, College of Dentistry, Princess Nourah bint Abdulrahman University P.O. Box 84428, Riyadh 1167, Saudi Arabia. Investigation, resources, writing (original draft preparation).; e Dental Intern, College of Dentistry, Princess Nourah bint Abdulrahman University P.O. Box 84428, Riyadh 1167, Saudi Arabia. Investigation, resources, writing (original draft preparation).; f Academic Researcher, Health Sciences Research Center, Princess Nourah bint Abdulrahman University P.O. Box 84428, Riyadh 1167, Saudi Arabia. Validation, investigation, data curation.; g Medical Microbiologist, Department of Biology, College of Sciences, Princess Nourah bint Abdulrahman University P.O. Box 84428, Riyadh 1167, Saudi Arabia. Validation, investigation, data curation, visualisation.

**Keywords:** Enterococcus faecalis, irrigant, root canal irrigation, sodium hypochlorite, oxygen

## Abstract

**Purpose:**

To evaluate the antimicrobial effect of a new active oxygen fluid (Blue**®**m) as a root canal irrigant against Enterococcus faecalis compared to sodium hypochlorite (NaOCl).

**Material and Methods:**

Forty-five extracted single-canaled human teeth were selected, received root canal preparation, autoclaved, and contaminated with *Enterococcus faecalis*. The specimens were randomly allocated into three groups: Group (A) served as the negative control, receiving irrigation with saline (n = 15); Group (B) was irrigated with 5.25% NaOCl (n = 15); and Group (C) was irrigated with 10 mL of Blue**®**m (n = 15). Microbial sampling from the root canals was performed before and after irrigation. The difference between the pre-irrigation and post-irrigation colony-forming units (CFU/mL) was calculated. The data was analysed using a one-way ANOVA followed by post-hoc Tukey tests. The significance level was set at 5%.

**Results:**

Blue**®**m statistically significantly reduced the bacterial load compared to saline (p = 0.009), but NaOCl was most effective, outperforming both (p < 0.0001).

**Conclusion:**

Irrigation with Blue**®**m demonstrated antibacterial efficacy against *Enterococcus faecalis*, but it was not as effective as NaOCl.

The purpose of root canal therapy is to prevent or treat periapical disease. A successful root canal treatment requires combining mechanical shaping with effective chemical preparation that can reach into the anatomical complexities of the root canal system.^19^ A wide variety of materials and techniques focusing on the chemical eradication of root canal infection have been proposed and advocated. Despite the dynamic and diverse nature of endodontic microbiota, *Enterococcus faecalis* (*E. faecalis*) is considered to be one of the common microorganisms found in both primary and secondary root canal infections.^31^ This facultative anaerobic gram-positive coccus is notable for its ability to form biofilms in glucose-deprived environments,^22^ its high adaptability, and its resistance to antimicrobial agents. *E. faecalis* achieves this through several unique characteristics: it produces collagen-binding proteins, can survive in alkaline pH conditions,^30^ and withstands long periods of starvation by obtaining nutrients from hyaluronan via the enzyme hyaluronidase.^18^

Endodontic irrigants should ideally have comprehensive antimicrobial properties in addition to biocompatibility, as they may come into contact with periodontal tissues during treatment.^7^ Due to its ability to dissolve organic tissue and its potent antibacterial properties, sodium hypochlorite (NaOCl) is currently the most recommended and commonly used endodontic irrigant.^11^ However, NaOCl’s elevated cytotoxicity is associated with clinical accidents should the irrigant extrude beyond the confinement of the root canal into the surrounding vital tissues.^1^ This can result in differing degrees of tissue necrosis, severe pain, oedema, and diffuse ecchymosis.^14,16^

Over the past decade, active oxygen agents have been used for bleaching and added to over-the-counter tooth-whitening toothpastes. Using these toothpastes was associated with a reduction in periodontal disease and anaerobic bacteria.^23^ Recently, a new active oxygen product, Blue**®**m (Wapenveld, Netherlands), was introduced to the market to accelerate oral wound healing after oral surgery and periodontal treatment.^10,33^ This product contains oxygen-releasing compounds such as sodium perborate, sodium percarbonate, and mel extract, which is derived from the filtration and concentration of honey.^5,33^ It relies on active oxygen to accelerate tissue re-modelling. Additionally, it contains xylitol and lactoferrin, which promote bone growth.^5,33^ Previous research demonstrated that the use of an active oxygen mouthwash significantly decreased the Gram-negative bacteria *Porphyromonas gingivalis* and *Streptococcus mutans*.^29,32^ At low concentrations, Blue**®**m can induce cell proliferation,²³ and promote gingival healing following oral surgery.^21,27^ This suggests that Blue**®**m has a low cytotoxicity level, which could be a significant advantage when used as a root canal irrigant.

The effect of Blue**®**m on endodontic microbiota and the possibility of using it as a root canal irrigator have not been investigated. Consequently, the aim of this study was to compare the antimicrobial efficacy of oxygen fluid (Blue**®**m) and NaOCl as root canal irrigants against *E. faecalis*. The null hypothesis was that the antibacterial effects of Blue**®**m and NaOCl are identical.

## MATERIALS AND METHODS

This ex vivo study was approved for exemption, according to the Internal Review Board at Princess Nourah bint Abdulrahman University (IRB no. 22-0443). The sample size calculation was performed using G*Power 3.1 software (Heinrich-Heine-Universität, Düsseldorf, Germany). An effect size (f) = 0.5 was considered, with an estimated power set at 0.80 and a prob-ability of Type 1 error α of 0.05.^9^

### Sample Preparation

Forty-five extracted single-canal human teeth were selected. Exclusion criteria included teeth with curved canals, external and internal resorption, double canals, and calcified canals. The crowns were removed, and root lengths were standardised at 14 mm. Canals were prepared using rotary nickel-titanium instruments: ProTaper Universal (Dentsply Sirona, Charlotte, NC, USA) until file size F3, followed by Profile 35/0.04 files according to the manufacturers’ instructions. The files were used with a 6:1 reduction contra-angle connected to a rotary electric Endo motor (X-smart, Dentsply Sirona, Charlotte, NC, USA). Canals were irrigated with 5 ml of 5.25% NaOCl (Pharma Vitality, Riyadh, KSA) between every file. A final rinse of 5 ml of 5.25% NaOCl with 3 ml of 17% EDTA for 1 min in alternation with 5 ml of saline was performed.

To avoid external microbial contamination, the external surface of the specimens was coated with two layers of nail varnish, and the apex was sealed with glass-ionomer cement before autoclaving. The teeth were autoclaved in a steam steriliser (Steris Amsco Century Prevac Steam Sterilizer, v-148h, United States) at a temperature of 132°C with a pressure of 29 psi for a period of 14 min.

### Bacterial Contamination

Standard strains of *E. faecalis *(American Type Culture Collection 29212) were prepared and cultured in brain heart infusion broth (BHI) and incubated for 24 h. Each tooth was placed in 300 microlitres of the BHI suspension containing bacteria. The teeth were incubated in an incubator shaker for a week at a temperature of 37°C, and the BHI-containing bacteria were renewed every 2 days under sterile conditions.

### Microbial Sampling

Under strict aseptic conditions, microbial sampling was performed twice: before and after irrigation. For all the groups, the first sampling was done by flooding the canal with sterile 0.05 mol/L phosphate-buffered saline (PBS) and then inserting a size 40 H-file (MANI, Tochigi, Japan) within the canal, 1 mm short of the working length, and circumferentially filing for 15 s. The canal contents were absorbed into ProTaper Universal F3 paper points inserted into the canal for 60 s and then transferred into test tubes that contained 1.0 ml of PBS. The contents of each canal were serially diluted and plated on BHI agar plates.

For the second microbial sampling, the specimens were randomly allocated into four groups: Group (A) served as the negative control, receiving irrigation with 10 ml of saline (n = 15); Group (B) served as the positive control, receiving 10 ml of 5.25% NaOCl (n = 15); and Group (C) was irrigated with 10 ml of Blue**®**m (n = 15).

To simulate clinical conditions, all irrigation procedures were performed using a syringe with a 30-gauge side-vented irrigation needle inserted 1 mm short of the full working length. The procedure was performed under strict aseptic conditions at a flow rate of approximately 10 ml per min.

In a manner identical to the first sampling, the canal contents were absorbed, serially diluted, and plated on agar plates. The agar plates for both samplings were incubated for 24 h. Colonies were then counted from magnified images. The difference between the pre-irrigation and post-irrigation colony-forming units (CFU/ml) was calculated, and log₁₀(x+1) transformation of values was applied.

### Statistical Analysis

Data were analysed using SPSS software version 22 (SPSS, Chicago, IL, USA). Data was analysed using a one-way analysis of variance ANOVA followed by Tukey’s post-hoc tests for multiple comparisons. A significant level of p ≤ 0.05 was selected.

## RESULTS

**Fig 1:**
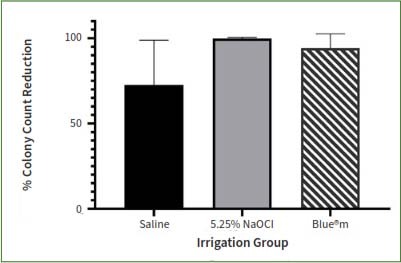
Reduction in colony-forming unit values (difference between pre- and post-irrigation CFU/ml) with different irrigants.

The mean differences between pre- and post-irrigation samplings were 0.862 ± 0.709 for the saline group, 2.871 ± 0.6415 for the NaOCl group, and 1.554 ± 0.547 for Blue**®**m (Fig 1). While Blue**®**m resulted in a statistically significant log₁₀ reduction compared to saline (p = 0.009), NaOCl achieved the greatest reduction, statistically significantly outperforming both Blue**®**m and saline (p < 0.0001) (Table 1).

**Table 1:** Mean log_10_ values for colony-forming units pre- and post-irrigation with the different irrigants

Irrigant	Sampling	Mean ± Std. deviation	95% Confidence interval	
			Lower limit	Upper limit
Saline	Pre	5.988 ± 0.342	5.798	6.177
	Post	5.125 ± 0.651a	4.764	5.486
5.25% NaOCl	Pre	5.968 ± 0.537	5.670	6.265
	Post	3.096 ± 0.373b	2.890	3.303
Bluem	Pre	6.094 ± 0.158	6.006	6.181
	Post	4.540 ± 0.470c	4.280	4.800

* Different letters indicate significant difference. * p value set at 0.05.

## DISCUSSION

Due to its high adaptability and resistance to antimicrobial irrigants, *E. faecalis* is common in endodontic secondary infections. Even if they undergo a phase of starvation, *E. faecalis* cells can form biofilm on human dentin, confirming their role in persistent apical periodontitis.^25^ Consequently, it is routinely selected as a test subject for new therapeutic compounds in root canal therapy.^24^ Concentrations ranging from 0.5% to 6% of NaOCl are used in endodontic treatment to disinfect root canals.^17^ The present results showed that 5.25% NaOCl was the most effective irrigant in reducing the bacterial load, followed by Blue**®**m, with saline being the least effective among the three. A previous study evaluating the efficacy of different NaOCl concentrations (0.5%, 2.5%, and 5.25%) against *E. faecalis* within prepared root canals and their dentinal tubules also showed that 5.25% NaOCl is the most effective irrigant solution tested, followed by 2.5% NaOCl.^3^ Another study found that only 5.25% NaOCl can effectively disaggregate and eradicate the *E. faecalis* biofilm.^17,25^

Sodium hypochlorite possesses characteristics of an ideal root canal irrigant, such as the ability to dissolve organic tissue, strong antimicrobial efficacy, a long shelf life, and affordability.^2^ It can dissolve amino and fatty acids, leading to the formation of chloramine, salt, and soap. Both these saponification and chloramination reactions lead to an elevated pH that exceeds 11, which in turn alters cytoplasmic membrane integrity by inhibiting enzymes and distorting nutrient transport, leading to bacterial cell death.^13^

Unfortunately, such effects can extend beyond bacterial cells. The main disadvantage of using NaOCl, especially at a high concentration, is the possibility of severe clinical repercussions associated with its inadvertent extrusion into the periradicular tissues during root canal irrigation due to its cytotoxicity.^15^ The term ‘NaOCl accident’ has been used to describe the resulting local necrosis of the oral mucosa, extensive swelling, hematoma, and possibility of neurological defects.^16^

Consequently, the pursuit of viable alternatives is of interest. Recently, Blue**®**m mouthwash has been promoted as a high-concentration active oxygen solution that stimulates and supports the healing process following inflammation. One study revealed that its use in patients receiving dental implants was associated with a significant decrease in Gram-negative *Porphyromonas gingivalis*.^29^ It showed comparable antibacterial efficacy to chlorhexidine mouthwash. Blue**®**m had a substantial antimicrobial effect against *Streptococcus mutans* at concentrations of 25% and 50%, according to a confocal microscopy study. At low concentrations, active oxygen was reported to control microbial colonisation, immunological response, and cell function with no adverse effects on fibroblasts.^32^

In the present study, Blue**®**m showed antibacterial efficacy against *E. faecalis*. This may be attributed to the presence of sodium perborate, a bleaching agent found in Blue**®**m.^6,35^ Under the typical warm and humid conditions of root canals, sodium perborate releases oxygen (O_2_), which can rapidly kill bacterial cells.^26^ Bluem fluid also contains sodium percarbonate, which decomposes into low concentrations of hydrogen peroxide (H_2_O_2_).^6^ Hydrogen peroxide is a broad-spectrum antimicrobial agent that is effective against highly resistant, dormant bacteria.^35^

Although Blue**®**m demonstrated antibacterial efficacy against *E. faecalis*, the present findings show that it was not as effective as NaOCl. Therefore, the null hypothesis was rejected. NaOCl showed superior efficacy compared to Blue**®**m. That said, it may be of interest to explore the effect of combining Blue**®**m with low, less cytotoxic concentrations of NaOCl on bacterial biofilm.

The active oxygen effect of Blue**®**m may also help boost adjunct activation techniques such as ultrasonic energy or Er,Cr:YSGG laser-activated irrigation to better eliminate mature biofilms.^4,12^ This potential benefit merits further exploration.

Endodontic infections are mediated by biofilms, which develop from surface-adherent bacterial cells and consist of a multicellular microbial community embedded in an extracellular matrix.^20^ The endodontic microbiota is highly diverse, encompassing various bacterial species.^28^ Although the 1-week incubation period used in the study was likely sufficient for the formation of *E. faecalis* biofilm,^34^ the results may still be overestimated.^8^ Additionally, the study did not investigate the penetration of bacteria into the dentinal tubules. Further research using a biofilm model that includes mixed bacterial species grown on dentine is recommended.

## CONCLUSIONS

Within the limitations of this study, Blue**®**m demonstrated antibacterial efficacy against *E. faecalis*, suggesting its potential as a viable alternative to traditional irrigants. However, its antimicrobial efficacy did not match that of NaOCl which remains superior in root canal disinfection. Future studies, however, could explore the potential benefits of combining Blue**®**m with lower, less cytotoxic concentrations of NaOCl to enhance its antimicrobial properties.

## Data Availability

The data sets generated and analysed in this study are available as a supplementary file directly from the corresponding author.
